# Easy Preparation of Liposome@PDA Microspheres for Fast and Highly Efficient Removal of Methylene Blue from Water

**DOI:** 10.3390/ijms222111916

**Published:** 2021-11-03

**Authors:** Vincenzo De Leo, Anna Maria Maurelli, Chiara Ingrosso, Fabio Lupone, Lucia Catucci

**Affiliations:** 1 Department of Chemistry, University of Bari Aldo Moro, Via Orabona 4, 70126 Bari, Italy; anna.maurelli@uniba.it (A.M.M.); luponefabio@libero.it (F.L.); 2 National Research Council of Italy-Institute for Physical and Chemical Processes (CNR-IPCF S.S. Bari), c/o Department of Chemistry, University of Bari Aldo Moro, Via Orabona 4, 70126 Bari, Italy; c.ingrosso@ba.ipcf.cnr.it

**Keywords:** liposomes, polydopamine, Liposome@PDA, methylene blue, adsorption, water remediation, adsorption kinetics, adsorption thermodynamic, wastewater, reusability

## Abstract

Mussel-inspired chemistry was usefully exploited here with the aim of developing a high-efficiency, environmentally friendly material for water remediation. A micro-structured material based on polydopamine (PDA) was obtained by using liposomes as templating agents and was used for the first time as an adsorbent material for the removal of methylene blue (MB) dye from aqueous solutions. Phospholipid liposomes were made by extrusion and coated with PDA by self-polymerization of dopamine under simple and mild conditions. The obtained Liposome@PDA microspheres were characterized by DLS and Zeta potential analysis, TEM microscopy, and FTIR spectroscopy. The effects of pH, temperature, MB concentration, amount of Liposome@PDA, and contact time on the adsorption process were investigated. Results showed that the highest adsorption capacity was obtained in weakly alkaline conditions (pH = 8.0) and that it could reach up to 395.4 mg g^−1^ at 298 K. In addition, adsorption kinetics showed that the adsorption behavior fits a pseudo-second-order kinetic model well. The equilibrium adsorption data, instead, were well described by Langmuir isotherm. Thermodynamic analysis demonstrated that the adsorption process was endothermic and spontaneous (ΔG^0^ = −12.55 kJ mol^−1^, ΔH^0^ = 13.37 kJ mol^−1^) in the investigated experimental conditions. Finally, the applicability of Liposome@PDA microspheres to model wastewater and the excellent reusability after regeneration by removing MB were demonstrated.

## 1. Introduction

Water pollution is unfortunately a widespread problem on a global scale. The causes of the phenomenon are numerous and are linked to different sectors, including the industrial, agricultural, and civil sectors. The problem is destined to increase over the years due both to the worsening of pollution caused by an unsustainable development model and to the impoverishment of water sources due to climate change. Therefore, the sustainable management of water represents one of the most urgent challenges at a global level, as mentioned in the *Sustainable Development Goals* of *2030 Agenda for sustainable development* adopted by United Nations in 2015 [1].

Methylene blue (MB) is a cationic dye commonly used in textile and paper industries, as well as in pharmacological applications, as a drug [2,3,4,5]. Mainly due to industrial uses it is released into water bodies, where it is difficult to degrade due to its stable aromatic molecular structure [6]. MB thus dispersed in significant concentrations can have negative effects on human health (skin irritation, gastrointestinal disorders, cyanosis, tachycardia, methemoglobinemia, dyspnea, etc.) [7] and on the ecosystem, increasing the carbon content in water and altering light capacity to penetrate water bodies and, thus, interfering with photosynthesis. Therefore, numerous experimental strategies were put in place to obtain an effective removal of MB from water, which involved the use of the most disparate methods and materials. Among the main ones, separation materials, adsorption materials, catalytic materials, and photothermal materials can be included [8]. Adsorption methods are very popular due to their effective pollutant removal effect associated with low cost, less secondary pollution, and extreme ease of use [5]. The adsorption method relies on various inorganic and organic adsorbent materials, obtained synthetically or by chemical modification of matrices and wastes of natural origin such as activated carbon, zeolite, chitosan, clay minerals, plant wastes, etc. [9]. The adsorption capacity and the adsorption mechanism (Van der Waals forces, electrostatic interaction, coordination interaction, and hydrogen bonding) are regulated both by the surface area and by the chemical properties of the surface, although these materials can be structured in very diverse ways as nanoparticles, microspheres, nanofibers, and sponges [8]. The major research efforts are aimed at obtaining materials with high adsorption performance and are capable of operating in a very short time.

Mussel-inspired chemistry has developed since the 1980s, with studies by Waite et al. concerning the strong adhesion properties of byssus from mussels to a variety of materials. They isolated from the byssus a protein containing large amounts of lysine, 3- and 4-hydroxyproline, and 3,4-dihydroxyphenylalanine (DOPA) [10]. Subsequent studies showed that the catechol groups of DOPA are mainly responsible for the adhesion properties of this material, in addition to lysine, which plays a cooperative role in the underwater adhesion processes [8,11]. Later, Lee et al. demonstrated that dopamine (DA), a DOPA derivative that contains catechol and amino groups, can self-polymerize to form polydopamine (PDA) under mild conditions and thus adhere to a large variety of organic and inorganic materials [12]. On the basis of this finding, PDA and its derivatives have begun to find application in the most disparate fields of research, from the biomedical field to the energy, sensing, and environmental ones [13]. In the field of water treatment, PDA has found applications in the removal of heavy metals and organic pollutants, due to its excellent adsorbing capacity [8,14], as well as in pathogenic microorganisms, thanks to its antibacterial and antifungal effects [15]. PDA, in fact, has numerous catechol and amino groups, as well as aromatic moieties that can bind both organic and inorganic pollutants through different types of interactions, including hydrogen bonding, electrostatic and π-π interactions, and coordination or chelation bonding [13]. A variety of PDA-based adsorbent materials have been designed and developed, including composites with other organic and inorganic materials, in the form of particles or surfaces of various morphologies. However, adsorbents made only of PDA generally exhibit low adsorption capacity and poor stability. Composite materials, on the other hand, to improve their performance, often require complicated and difficult-to-scale procedures.

Improving the performance of these adsorbent systems is conceivable by preparing PDA-based structures with a high surface–volume ratio and improved colloidal stability. Systems with these characteristics were recently obtained in a relatively simple way by growing a PDA shell on the surface of liposomes and were proposed for applications in the biomedical field [16,17]. Liposomes are polymolecular aggregates of lipid molecules (mostly phospholipids) that auto-assemble in water in a supramolecular architecture, assuming a spherical shape. Their composition and structural organization are very similar to those of biological membranes, with the lipids paired to form a double layer closed on itself, trapping part of the solvent in which they are dispersed [18]. If the lipids used are of natural origin, such as phosphatidylcholine (POPC), the liposomes are biocompatible and biodegradable, as widely recognized [18,19]. Liposomes also find application in a wide variety of fields: reconstitution of membrane proteins, delivery of drugs and fluorescent probes, functionalization of surfaces for biomedical applications, synthesis of nanomaterials, and much more [20,21,22,23,24]. In addition, liposomes were used as colloidal templates for the preparation of nano and micro materials with controlled structure and functionality, exploiting either the aqueous core, such as a microreactor, or the surface for the controlled polymerization of different precursors [25,26].

**Figure 1:**
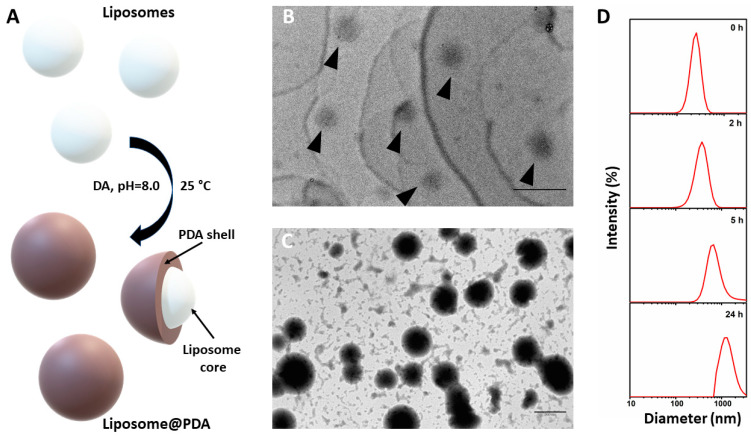
(**A**) Sketch of the Liposome@PDA microspheres preparation. (The schemes are not drawn to scale). TEM images of (**B**) as prepared liposomes (indicated by arrowheads, scale bar 500 nm) and (**C**) Liposome@PDA microspheres after 24 h of DA polymerization (scale bar 500 nm). (**D**) Hydrodynamic diameter of microspheres obtained by DLS measurements at diverse times of polymerization of DA.

In this work, phospholipid-based liposomes were used as templating agents to obtain Liposome@PDA microspheres, inducing DA polymerization in mild conditions (pH 8.00 and in an air atmosphere) without the use of organic solvents or hard templating cores, unlike other works previously reported [2,5]. The DA monomer polymerization in PDA takes place on the surface of the liposomes to give core-shell structures (Liposome@PDA, Figure 1A). This adsorbent system is assumed to be biocompatible and biodegradable as its building blocks. POPC liposomes and PDA polymer are in fact considered largely biodegradable and biocompatible as demonstrated by a large scientific literature [19,27,28]. Furthermore, Liposome@PDA microspheres were obtained without the use of organic solvents through a totally green strategy. Therefore, there should be no concerns related to their accidental release into the environment. Liposome@PDA microspheres can be easily suspended in water during the adsorption process and can also be easily removed by spontaneous sedimentation or centrifugation in few seconds. The utilization of these PDA-modified vesicles for the removal of environmental pollutants has not been previously reported.

## 2. Results and Discussion

### 2.1. Preparation and Characterization of Liposome@PDA Microspheres

Liposome@PDA microspheres were obtained by polymerization of DA under mild conditions, in aqueous solutions at pH 8.00 and exposed to air under gentle stirring (Figure 1A). Interestingly, the polymerization in absence of liposomes in these conditions was very poor and limited to the deposition of a thin layer of PDA on the walls of the reaction vessel, while the solution remained almost clear (Figure S4). On the contrary, in presence of liposomes, the solution gradually turned dark over the course of hours. The yield of polymerization under the experimental conditions was in any case low and reached a value of about 20% after 24 h at room temperature. TEM images show liposomes as low-contrast, 212 ± 54 nm-sized, spherical-shaped nanostructures (Figure 1B). After 24 h of DA polymerization, TEM images revealed higher contrast spherical nanostructures, 439 ± 80 nm in size (Figure 1C), ascribed to Liposome@PDA microspheres, showing the retention of the pristine spherical morphology of the liposomes used as template agents and a significant increase of the diameter. Overall, the appearance of Liposome@PDA microspheres resembles that of other previously prepared PDA-based structures, with the boundaries of some of them difficult to identify, probably due to aggregation phenomena [2,29]. This evidence allowed one to infer the growth of the polymer around the surface of the liposomes used as templates. This hypothesis was also supported by DLS measurements, which were used to monitor the polymerization process over time (Figure 1D). The liposomes were obtained by extrusion with a 200 nm membrane (see paragraph 2.2). The mean diameter measured at zero time of polymerization was in fact 237 nm. The size distribution showed a widening and a shift to higher values during the polymerization of DA, indicating a progressive growth of the PDA layer and an increase in the degree of dispersion. After 24 h, the size reached the micrometric scale, but no other peaks emerged from the analysis, suggesting that there was no auto-polymerization of the DA to form PDA-only particles. As expected, the Liposome@PDA nanostructures estimated from TEM micrographs are smaller than those measured by DLS, because the hydrodynamic diameter achieved from DLS traces is generally larger than the average particle size. Moreover, the TEM images showed the formation of some coalesced spherical structures likely originated from polymerization of DA around two or more liposomal nuclei (Figure 1C), thus affecting DLS measurements.

**Figure 2:**
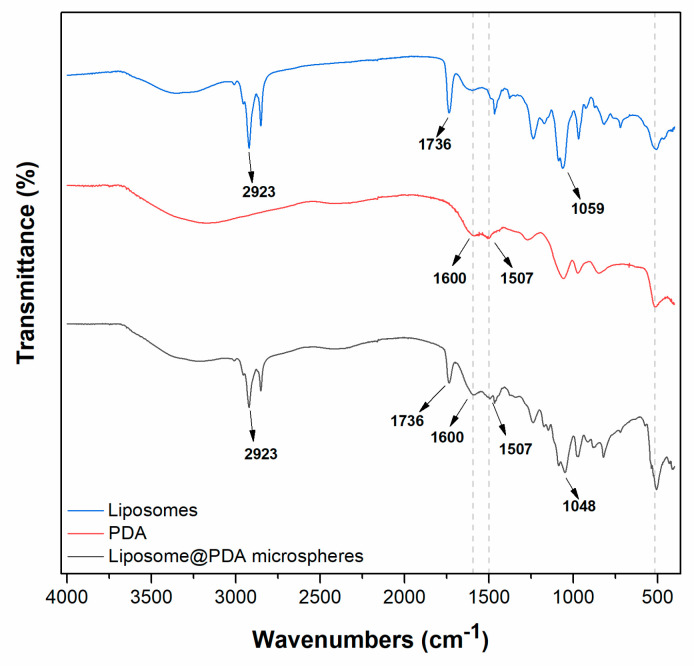
FTIR spectra of liposomes, PDA, and Liposome@PDA microspheres.

FTIR spectra of liposomes, PDA and Liposome@PDA microspheres are displayed in Figure 2. At high wavenumbers, the spectrum of liposomes contains the signals from the lipid hydrocarbon chains, mainly from C-H stretching vibrations (narrow peaks at 2923 cm^–1^ e 2850 cm^–1^). Peaks related to the lipid polar head groups appear at lower wavenumbers, including the peak at 1736 cm^–1^ due to the C = O ester stretching and at 1059 cm^–1^ due to the C–O ester stretching [30,31]. In the PDA spectrum, the wide absorption band at 3190 cm^–1^ is due to stretching vibrations of the phenolic O-H and N-H groups, while the peaks at 1507 cm^–1^ and 1616 cm^–1^ are accountable to N-H bending vibration and to the overlapping of the C = C resonance vibration of the aromatic ring, respectively [2,5]. The successful coverage of liposomes with PDA was confirmed by the presence of the signals of both components in the spectrum of Liposome@PDA microspheres (see eye guides in Figure 2). The peak of C–O ester stretching is slightly shifted to 1048 cm^−1^. Although in Liposome@PDA microspheres the PDA shell surrounds the liposomes, as suggested by TEM investigation, it is reasonable to find the infrared signals of both the lipids and PDA in the spectra, because the penetration depth of the evanescent wave in ATR measurements is between 0.5–5 μm, depending on the spectrum region.

### 2.2. Adsorption Experiments of MB Onto Liposome@PDA Microspheres

**Figure 3:**
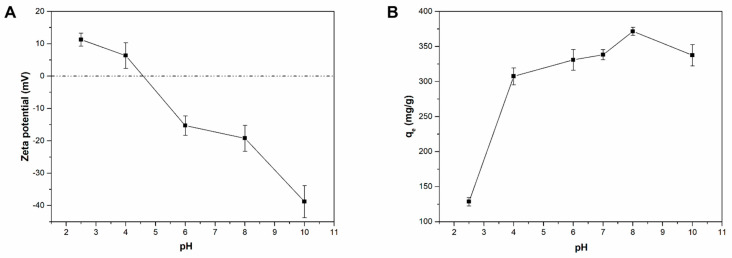
(**A**) Zeta potentials of Liposome@PDA microspheres at different pH. (**B**) Effect of pH on dye adsorption on Liposomes@PDA microspheres. MB concentration: 3.2 mg L^−1^; mass of Liposome@PDA microspheres: 0.06 mg; temperature: 298 K; equilibration time: 90 min.

Figure 3A shows the Zeta potential values of the Liposome@PDA microspheres measured at different pH within the range 2.5–10.0. Liposome@PDA microspheres exhibited positive surface charge at acid pH values due to the protonation of the amino groups of the PDA coating. Zero point charge (PZC) of Liposome@PDA microspheres was calculated as pH 4.5 and at higher values the sign of the surface charge reversed, becoming markedly negative at alkaline pH due to the deprotonation of the phenolic groups on the PDA shell, a behaviour similar to that observed for PDA microspheres [2]. By monitoring the variation of the adsorption capacities of MB for a time long enough to reach an equilibrium condition (q_e_), in the same pH range in which the Zeta potential of Liposome@PDA microspheres was measured, it is evident that the pH has a crucial effect on the extent of the adsorption (Figure 3B). In strongly acidic conditions, i.e., when the surface charge exhibited by the Liposome@PDA microspheres is positive, the adsorption of the cationic molecules of MB was hindered by electrostatic repulsion, and the lowest q_e_ values were observed. The adsorption capacities progressively increased in the pH range 4.5–8.0 and reached the highest value of 371.7 mg g^−1^ at pH 8.0, when the Zeta potential was strongly negative, confirming that the electrostatic interaction is the main mechanism that drives the dye adsorption phenomenon. The slight decrease in adsorption capacity observed at pH 10.0 could be due to the fact that the PDA shell in a strong basic environment has been slightly broken [29], as well as the possibility of dye aggregation phenomena, which would lead to an inaccurate interpretation of the adsorption, as reported by other authors [32].

However, the q_e_ values were still considerable across the pH values that correspond to PZC, suggesting that the adsorption process of Liposome@PDA microspheres toward MB dye can be due to several mechanisms. Indeed, in addition to the electrostatic interaction between MB cations and the negatively charged surface of Liposome@PDA microspheres at alkaline pH, the following phenomena can also contribute to the adsorption, as suggested for similar PDA-coated systems: (i) π–π stacking interactions between MB and aromatic rings of PDA, as well as π-cation interaction between MB and aromatic rings of PDA; (ii) hydrogen bonding between the negatively charged oxygen group of PDA and the amine group of MB; and (iii) physical adsorption of MB molecules by the high surface area of Liposome@PDA microspheres [2,5,33].

**Figure 4:**
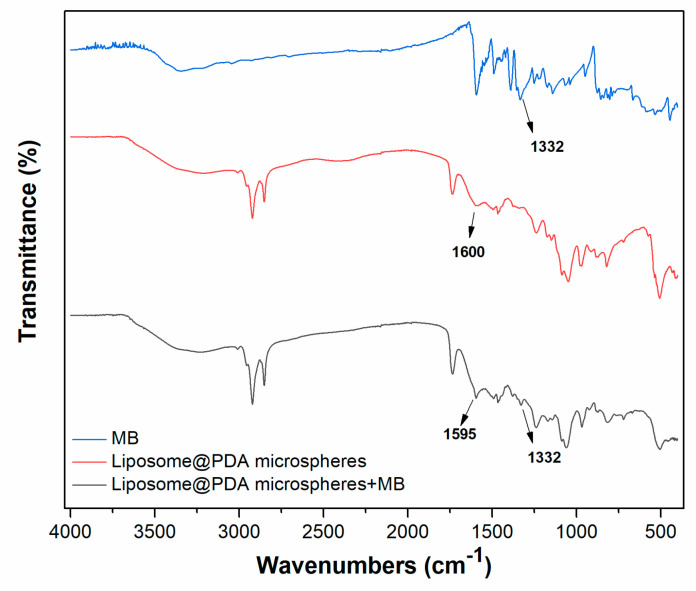
FTIR spectra of MB and Liposome@PDA microspheres before and after adsorption of MB.

In fact, as shown in Figure 4, the IR spectrum of the adsorbent changed after the adsorption of the dye. As reported by other authors, the most relevant effect seems to be the shift of the signal related to the -C = C- stretching from 1600 cm^−1^ to 1595 cm^−1^, suggesting a π-π stacking interaction involved in adsorption process, although it remains difficult to discern the contribution of a convolution with the relative signal of MB.

**Figure 5:**
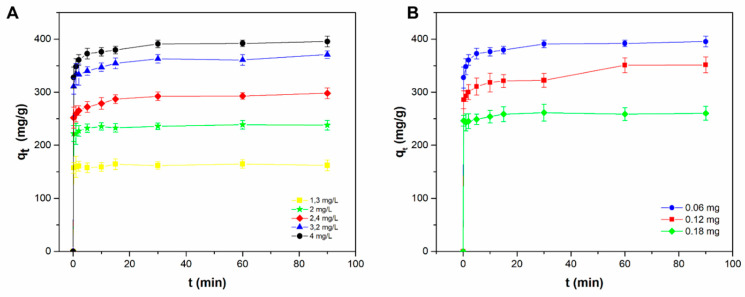
(**A**) Variation of MB adsorption capacity on Liposomes@PDA microspheres as a function of contact time as the initial dye concentration varies. Adsorbent mass: 0.06 mg; temperature: 298 K; pH = 8.0; equilibration time: 90 min. (**B**) Variation of MB adsorption capacity on Liposomes@PDA microspheres as a function of contact time at different mass of adsorbent Liposome@PDA. MB: 4 mg L^−1^; temperature: 298 K; pH = 8.0; equilibration time: 90 min.

In any case, in view of a real application, the subsequent adsorption tests were carried out at pH 8.00, which was the pH value at which the best adsorption performance of MB by Liposome@PDA microspheres was obtained. Figure 5A shows the effect of contact time on the adsorption capacity of MB on Liposome@PDA particles at different initial concentrations of dye, from 1.3 to 4 mg L^−1^, while dosage of Liposome@PDA adsorbent was kept constant. Experiments have shown that MB adsorption was very fast for all initial concentrations tested, with q_t_ values undergoing a dramatic increase already within the first minute. Subsequently, the adsorption progressively slowed down until equilibrium was reached. The adsorption equilibrium time was found to be about 35 min, and no significant changes in q_t_ values occurred over the next 55 min. The trend of the adsorption process is justified by the large number of sites available on the surface of the Liposome@PDA microspheres at the beginning of the experiment. As these sites are occupied, the adsorption slows down due to the repulsive interactions that occur between the MB molecules on the PDA surface, and between those on the surface and those in the bulk phase [2]. The adsorption process appeared to be dependent on the initial MB concentration. In particular, the q_e_ values progressively increased with increasing MB concentration. This experimental result can be explained admitting that the increased MB concentration in aqueous solution promoted a higher gradient between the solution and the Liposome@PDA microspheres surface, increasing dye mass transfer from the bulk phase to the microspheres as well as the MB adsorption capacity. In addition, at higher concentrations of MB, the possibility of a collision between the dye molecules and the adsorbent is statistically more probable, thus increasing the adsorption [21]. On the contrary, the saturation of the active adsorption sites led to a decrease of removal efficiencies (see Equation (2)) from 96.2% to 77.9% as the concentration of the dye increases.

As shown in Figure 5B, q_e_ value decreased with increasing Liposome@PDA amount from 0.06 to 0.18 mg, while the concentration of the MB was kept constant. In contrast, the removal efficiencies increased from 77.9% to 85.1% with increasing the amount of adsorbent. This phenomenon can be explained considering that larger dosage of Liposome@PDA microspheres can provide higher amount of adsorption sites, thus resulting in a decrease of the concentration of free MB in solution. Concurrently, the utilization rate of adsorption sites is decreased by the lower concentration of free dye, which leads to a low equilibrium adsorption capacity [34].

The maximum adsorption capacity at equilibrium for MB reached 395.4 mg g^−1^, a value higher than those of similar PDA-based adsorbent materials reported in literature (Table 1). The high q_e_ values measured can be justified in light of the peculiar characteristics of the Liposome@PDA microspheres. The spherical morphology associated with these structures gives them better performance in terms of mass diffusion and transport compared to conventional adsorbent materials [2,35]. Furthermore, the micrometric dimensions associated with a hollow structure determine a high specific surface area, leading to optimal contact conditions between the adsorbent material and the dye. Finally, the Liposome@PDA microspheres maintained an excellent dispersibility in water typical of the colloidal systems from which they originated.

### 2.3. Kinetic Studies of the Adsorption Process

Kinetic parameters provide important information on the dynamics of the adsorption processes. Usually, the mechanism of solid–liquid adsorption process is represented by four steps: (i) transport of adsorbate molecules from liquid bulk phase to the liquid film (boundary layer) that surrounds the adsorbent; (ii) subsequent transport to the outer surface of the adsorbent material (film diffusion); (iii) transfer of adsorbate molecules into the intraparticle cavity of adsorbent (intraparticle diffusion); and (iv) adsorption of adsorbate by the active sites of adsorbent material. The rate-controlling steps depend on second and third steps, i.e., film diffusion and intraparticle diffusion as the first step does not provide any involvement of adsorbent, and the fourth step is a very rapid process [4,7]. Three well known kinetic models were used in this study to analyze the adsorption kinetic data and precisely pseudo-first-order model, pseudo-second-order model, and intraparticle diffusion model, respectively expressed as Equations (1)–(3):

(1) $$\ln\left(q_{e}-q_{t}\right)=\ln\left(q_{e}\right)-k_{1}t$$

(2) $$\frac{t}{q_{t}}=\frac{1}{k_{2}q_{e}{}^{2}}+\frac{t}{q_{e}}$$

(3) $$q_{t}=k_{i}t^{1/2}+c$$

where *q_e_* and *q_t_* (mg g^−1^) are the adsorption capacities at the equilibrium and at the contact time *t* (min), respectively. *k_1_* (min^−1^) and *k_2_* (g mg^−1^ min^−1^) are the rate constants of the pseudo-first-order and pseudo-second-order kinetic models, respectively. *k_i_* (g mg^−1^ min^−0.5^) is the intraparticle rate diffusion constant.

**Figure 6:**
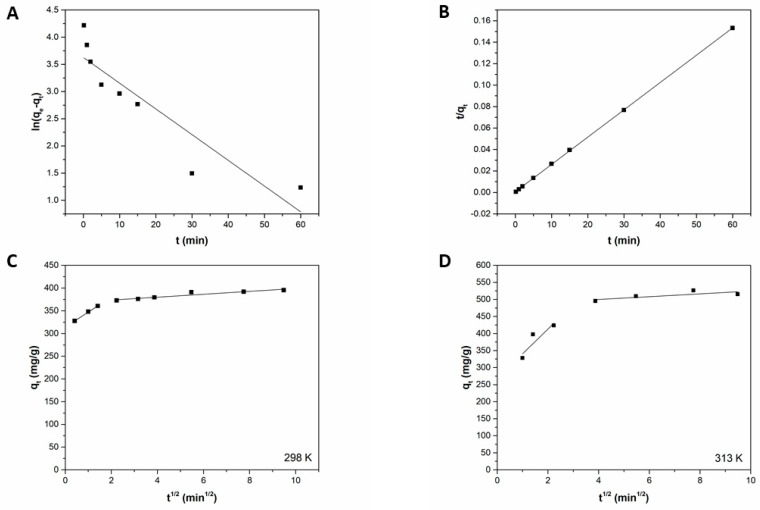
(**A**) Pseudo-first order, (**B**) pseudo-second order, and (**C**,**D**) intraparticle diffusion model at 298 K and 313 K, respectively, for MB adsorption onto Liposome@PDA microspheres.

Linear regression of the experimental data (Figure 6) provided the kinetic parameters reported in Table 2. Plots in Figure 6A,B show that the pseudo-second-order model clearly fits better with experimental data, possessing higher R^2^ value respect to pseudo-first-order model (Table 2). Furthermore, only the kinetic model of the pseudo-second-order provides a calculated q_e_ value very close to the experimental one. This outcome suggests that the adsorption process of MB onto Liposome@PDA microspheres was controlled by chemisorption [41]. Figure 6C shows the multilinear plot that corresponds to the various steps of the adsorption process studied through the Weber intraparticle diffusion model. The observed multilinearity and the absence of a straight line passing through the origin suggest that intraparticle diffusion is not the rate-limiting step of the process [3]. The graph shows that at least two steps take place during the adsorption of MB by the Liposome@PDA microspheres. The first corresponds to film diffusion and the second to intraparticle diffusion. The slope of the first stretch appears higher than that of the second, indicating a slower and more gradual process for intraparticle diffusion [2]. This is probably because the dye molecules meet a smaller free path as they diffuse into the internal structure of the PDA, encountering smaller and smaller pores [3]. Finally, the R^2^ values in Table 2 show the excellent applicability of the model to the experimental data. It was reported that with increasing temperature, the pore diffusion in adsorbent materials also increases, and as an outcome, the intraparticle diffusion rate enhances [42]. Indeed, the Weber intraparticle diffusion model applied to the experimental data obtained at 313 K (Figure 6D) showed that k_i_ values increased with solution temperature (Table 2).

### 2.4. Adsorption Isotherms

**Figure 7:**
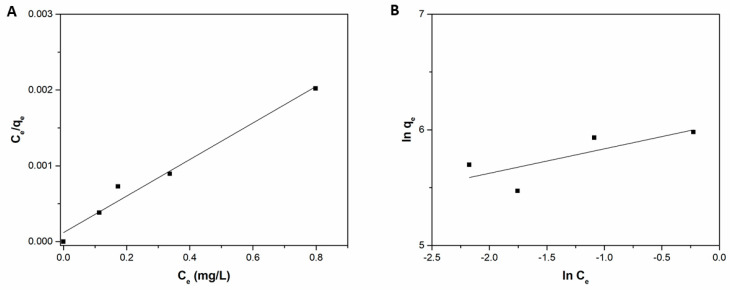
(**A**) Langmuir and (**B**) Freundlich isotherms for the adsorption of MB onto Liposome@PDA microspheres.

The adsorption isotherms were studied in order to obtain information on the surface properties of the adsorbent material and then on the behaviour of the solid-solution system. In fact, the adsorption isotherms provide information on the distribution of the adsorbate molecules between the solid and liquid phases, when the adsorption process is in an equilibrium state. The experimental data related to the adsorption of MB by Liposome@PDA microspheres in equilibrium conditions were fitted according to Langmuir and Freundlich models. Langmuir’s is a well-known isothermal adsorption model for the study of solid-solution systems, and it is based on the assumption that the adsorption process involves the formation of a monolayer and that the surface of the adsorbent material is homogeneous. In contrast, the Freundlich model predicts that the adsorbent surface is heterogeneous and that a multilayer adsorption process takes place. The following Equations in linear form can describe the two models:

(4) $$\frac{C_{e}}{q_{e}}=\frac{1}{q_{0}}C_{e}+\frac{1}{K_{L}q_{0}}$$

(5) $$\ln\left(q_{e}\right)=\ln\left(K_{F}\right)+\frac{1}{n}ln(C_{e})$$

where *K_L_* and *q_0_* are the Langmuir adsorption constant and the adsorption capacity, respectively, while *K_F_* and *n* are the Freundlich constant and the heterogeneity constant, respectively. Plots of both Equations (4) and (5) are shown in Figure 7, and the relative parameters are shown in Table 3. Although the Freundlich model returned a value of 1/n between 0 and 1, which corresponds to a favourable adsorption process, it correlates poorly with the experimental data (R^2^ = 0.5899). The latter are better suited to the Langmuir model, which shows a value of R^2^ close to unity, much higher than that relating to the Freundlich isotherm. Furthermore, the q_0_ value of the Langmuir model was very close to experimental data, indicating that Langmuir model was more suitable to describe the adsorption process of MB onto Liposome@PDA microspheres. The separation factor *R_L_* is another important characteristic of the Langmuir isotherm and can be calculated by the following Equation:

(6) $$R_{L}=\frac{1}{1+K_{L}C_{0}}$$

where *C_0_* is the initial MB concentration. The *R_L_* value indicates the nature and the feasibility of the adsorption process: irreversible (*R_L_* = 0), favourable (0 < *R_L_* < 1), linear (*R_L_* = 1), or unfavourable (*R_L_* > 1). Table 3 shows that *R_L_* is in the range 0.1220–0.0366 and therefore the adsorption for the system under consideration is favourable.

The specific surface area of Liposome@PDA adsorbent material was determined by the Langmuir adsorption isotherm model. It is worth noting that MB was adopted widely for this type of determination for various materials since this method is very simple, rapid, reliable, and cheap [43]. The specific surface area of Liposome@PDA microspheres at 298 K was determined to be 189.5 m^2^·g^−1^. This result is definitely superior to that of other similar systems (about an order of magnitude), justifying the high adsorption performance observed [2].

### 2.5. Adsorption Thermodynamics

**Figure 8:**
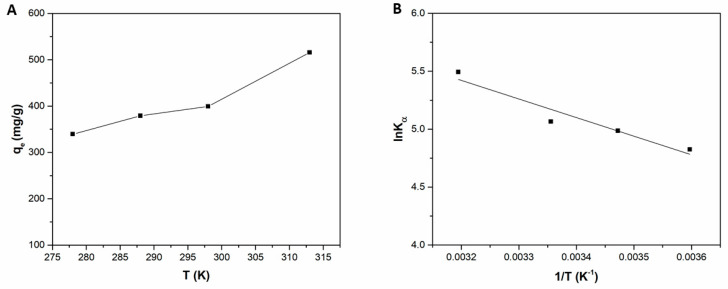
(**A**) Dependence of MB adsorption on temperature and (**B**) Vant’ Hoff plot of the MB adsorption onto Liposome@PDA microspheres.

The effect of temperature on the adsorption of MB onto Liposome@PDA microspheres was studied through batch adsorption experiment in the range 278–313 K. Figure 8A shows that the adsorption capacity increased from 339.4 to 515.6 mg g^−1^ with increasing temperature in the considered range. Therefore, the dye adsorption process by Liposome@PDA microspheres was endothermic, similarly to other PDA-based adsorbent systems [2]. The determination of the thermodynamic parameters was then performed to gain more information on the process. Thermodynamic parameters were calculated from the following Equations:

(7) $$lnK_{\alpha }=\frac{∆S^{0}}{R}-\frac{∆H^{0}}{RT}$$

(8) $$K_{\alpha }=\frac{q_{e}}{C_{e}}$$

(9) $$∆G^{0}=-RTlnK_{\alpha }$$

where *K_α_* (L g^−1^) is a constant in the Van’t Hoff Equation. *R* (8.314 Jmol^−1^ K^−1^) is the universal gas constant and *T* (K) the temperature of the system. The concentration of MB solution and the amount of adsorbed MB at the equilibrium are *C_e_* (mg L^−1^) and *q_e_* (mg g^−1^). ΔH° e ΔS° were determined from the Van’t Hoff plot (Figure 8B), respectively, from the slope and intercept. Table 4 shows that K_α_ values increased with increasing temperature, indicating that adsorption was favoured by increasing temperature. The ΔG° values were negative and therefore adsorption was a spontaneous phenomenon. The ΔG° values became progressively more negative to confirm that the increase of temperature favoured MB adsorption. The positive value of ΔH° is typical of an endothermic process. The positive ΔS° value reflects the affinity of the adsorbent material for the MB and suggests that the degree of disorder at the solid–liquid interface increases during the interaction of the dye with the Liposome@PDA microsphere surface, likely due to some structural changes in adsorbate and adsorbent, as previously reported [44].

### 2.6. Adsorption Experiments in Model Effluent and Reusability

**Figure 9:**
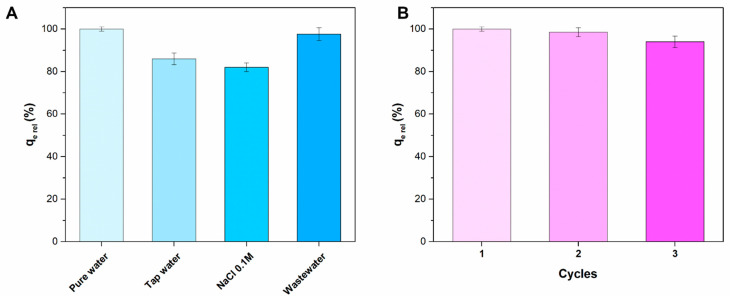
(**A**) Adsorption experiments in model effluent. (**B**) Adsorption performance and reusability of Liposome@PDA microspheres. Adsorbent mass 0.06 mg. MB concentration 4 mg/L. Temperature 298 K and equilibration time 90 min.

Adsorption experiments in solutions containing salts and in simulated wastewater were conducted to evaluate the applicability of Liposome@PDA microspheres in real contexts. Figure 9A shows the results obtained in tap water, NaCl 0.1 M solution, and simulated wastewater compared to those obtained in pure water. Solution cations are known to compete with MB for adsorption sites on the PDA surface, thereby decreasing the performance of the adsorbent materials [40,45]. The tap water used for the experiment contained various cations, including sodium, calcium, magnesium, and potassium in appreciable concentration (for a complete analysis of tap water see reference [46]). These cations probably caused the MB adsorption reduction to 86% of the value measured in pure water. Data obtained in NaCl 0.1 M confirmed a similar reduction in the adsorption capacity due to sodium cations. In synthetic wastewater prepared in accordance with the OECD guideline for the testing of chemicals, the adsorption capacity dropped to only 98% compared to the values obtained in pure water. In any case, the conducted experiments showed that the adsorption capacity of Liposomes@PDA microspheres towards MB remained high even in complex solutions, indicating the possibility of their use in real contexts. Afterwards, adsorption–desorption experiments were carried out to assess the regeneration and stability of Liposome@PDA microspheres for MB uptake. Methanol was used as eluents to desorb MB, as previously reported [40]. The regenerated Liposome@PDA microspheres were used in the next adsorption processes. As shown in Figure 9B, the sorbent retained good adsorption efficiency at more than 90% of the original q_e_ value, after three cycles. The observed little decrease might be caused by the loss of sorbent material during rinsing operation in addition to irreversible occupation of part of the adsorption sites, as hypothesized previously by several authors for similar PDA-based sorbent [38,40].

## 3. Materials and Methods

### 3.1. Reagents

Ethanol, methanol, cholesterol, the reagent grade salts for phosphate buffer solutions, 2-(3,4-dihydroxyphenyl)ethylamine hydrochloride (dopamine), and methylene blue were obtained from Merck Italy (Merck Life Science s.r.l., Milan, Italy). Lipoid E80 (LE80, approximately 80% soybean phosphatidylcholine) was from Lipoid (Lipoid, Ludwigshafen, Germany).

### 3.2. Liposome@PDA Microspheres Preparation

Naked liposomes were prepared with the extrusion technique [47]. Briefly, 9 mg of LipoidE80 and Cholesterol 10% w/w were dissolved in chloroform and dried under gentle nitrogen flux in order to obtain a thin lipidic film on the walls of the vial. Then, the organic solvent was completely removed under vacuum conditions for 24 h. The dried film was hydrated with 1 mL of potassium phosphate saline buffer (KPi 50 mM, pH 8.0) and vortexed to allow formation of the lipidic vesicles in the buffer. Finally, to obtain a uniform distribution of the vesicles, the sample was extruded with a mini-extruder (Avanti Polar Lipids, Birmingham, AL, USA), by using polycarbonate membranes with a defined porosity of 200 nm (11 times).

Liposome@PDA microspheres were obtained under weak alkaline conditions (pH = 8.0) involving atmospheric oxygen as oxidant. Briefly, DA was added to the liposome suspension at pH = 8.0 (DA 0.5 mg mL^−1^, liposomes 1.8 mg mL^−1^). The solution was left to react under stirring (100 rpm) for 24 h at 288 K and under air atmosphere. Finally, the solution was centrifuged for 3 min at 7000 rpm in order to collect the Liposome@PDA microspheres and rinsed with fresh buffer three times to remove unreacted monomer. For the PDA control batch, 5 mg of DA was added to 10 mL of buffer at pH 8, and left to react in the same conditions, as described before. The polymerization yield of DA was calculated from absorption spectra collected by a Cary 5000 ultraviolet-visible double-beam spectrophotometer (Agilent Technologies, Santa Clara, CA, USA). Specifically, it was evaluated by measuring the decrease of the characteristic absorption peak of DA at 280 nm, as the monomer polymerizes into PDA (Figure S1). As the hydrodynamic diameter of the vesicles increases during polymerization, the scattering in the absorption spectra also increases. Therefore, to calculate the polymerization yield, the contribution of scattering to the spectra was first evaluated through an interpolation with a third-degree polynomial curve (see dashed curve in Figure S1). Subsequently this contribution was subtracted from the absorption spectra to obtain the right concentration of DA.

### 3.3. Liposomes and Liposome@PDA Microspheres Characterization

The hydrodynamic diameter was determined by means of dynamic light scattering (DLS) analysis by using a Nanoparticle Size Analyzer Horiba Jobin Yvonne LB-550 (Horiba Jobin Yvon SRL, Milano, Italy). The Zeta potential of Liposome@PDA microspheres at different pH was measured by laser doppler electrophoresis (LDE) with a Nanosizer ZS (Malvern instruments, Malvern, UK) as in [48]. Transmission electron microscopy (TEM) analyses were performed by using a Jeol Jem-1011 microscope (JEOL USA, Inc., Pleasanton, CA, USA), operating at 100 kV and equipped by a high-contrast objective lens, a W filament as an electron source, with an ultimate point resolution of 0.34 nm. TEM images were acquired by a Quemesa Olympus CCD Camera of 11 Megapixels. Statistical analysis of the average size and size distribution of the achieved Liposome@PDA structures was performed by using the freeware ImageJ analysis program. Samples were prepared by depositing 2 µL of the aqueous suspension containing the bare or PDA-coated liposomes on a 300-mesh amorphous carbon-coated Cu grid, drying the excess solution with absorbent paper and then allowing it to dry completely in the air. Infrared spectra were collected by a spectrometer *Spectrum One* (PerkinElmer, Waltham, MA, USA). For all measurements, spectral resolution was 2 cm^–1^. The internal reflection element used for ATR measurements was a one-bounce 4 mm-diameter diamond microprism. Freeze-dried samples were analyzed by placing just enough powder to cover the crystal area and by using the pressure arm.

### 3.4. Adsorption Experiments

Batch adsorption experiments were carried out at 298 K and involved varying the pH between 2.5 and 10 to find the conditions that best gave adsorption performance. One aliquot of Liposome@PDA microspheres (0.06 mg) was added to 10 mL of MB 3.2 mg L^−1^ and left to interact under stirring (200 rpm) for 90 min. The amount of MB adsorbed per unit of mass of Liposome@PDA microspheres was calculated in terms of adsorption capacity (Equation (10)) and removal efficiency (Equation (11)), by evaluating the decrease of the absorbance peak of MB at 665 nm into the supernatant [2] (Figures S2 and S3):

(10) $$q_{t\;}=\frac{\left(C_{0}-C_{t}\right)*V}{m}$$

(11) $$R=\frac{\left(C_{0}-C_{t}\right)}{C_{0}}*100\;$$

where *C_0_* (mg L^−1^) is the initial concentration of MB, *C_t_* is the concentration of MB after *t* min of adsorption, V is the volume of the dye solution expressed in liters, while m is the mass of Liposome@PDA microspheres expressed in grams. In order to stop the adsorption process at the desired time, an aliquot of the suspension was centrifuged first for 30 s at 12,000 rpm to rapidly separate most of the adsorbent material, and then the supernatant was centrifuged for other 15 min.

With the pH value fixed at 8, the effect of the contact time on the adsorption process was explored, keeping constant the number of Liposome@PDA microspheres and changing the concentration of MB (1.3, 2, 2.4, 3.2, and 4 mg L^−1^). Then, the effect of the amount of the adsorbent material was investigated increasing the mass of PDA adsorbent material (0.06, 0.12, and 0.18 mg) at a fixed MB concentration (4 mg L^−1^).

To calculate the thermodynamic parameters, adsorption experiments were conducted at different temperatures (278, 288, 298, and 313 K) by adding 0.06 mg of Liposome@PDA microspheres to 10 mL of a MB solution (4 mg L^−1^) and evaluating the adsorption capacity after 90 min.

All experiments were performed in triplicate, and when not specifically indicated, the temperature was 298 K.

### 3.5. Evaluation of the Specific Surface Area of the Liposome@PDA Microspheres

The specific surface area of the Liposome@PDA microspheres was calculated with the following Equation [43,49,50]:

(12) $$\beta =a*S_{MAX}*N_{AV}$$

where *a* is the area of a molecule of MB (1.3 * 10⁻^1^⁸ m^2^ [51,52]). *S_MAX_* is the maximum level of adsorption, and *N_AV_* is the Avogadro’s number.

*S_MAX_* was derived from the linearized Langmuir Equation:

(13) $$\frac{\left[MB\right]}{S}=\frac{1}{S_{MAX}*K_{L}}+\frac{\left[MB\right]}{S_{MAX}}$$

where *S* was calculated performing the adsorption experiment with 0.06 mg of Liposome@PDA microspheres and different MB concentrations (1.3, 2.4, and 4 mg L^−1^):

(14) $$S=\frac{adsorbed\;MB\;moles}{g\;Liposome@PDA}$$

### 3.6. Adsorption Experiments in Model Effluent and Reusability of Liposome@PDA Microspheres

Adsorption experiments in real samples and synthetic wastewater were realized. In particular, the following solutions were tested: (1) tap water, (2) NaCl 0.1 M, and (3) synthetic wastewater. The experiments were carried out in triplicate and the results normalized with respect to a reference test carried out in pure water and in optimized conditions. The tap water analysis can be found at the following link [46]. Synthetic wastewater was prepared according to OECD guideline for the testing of chemicals as reported in [40]. Adsorbent mass was 0.06 mg, MB concentration 4 mg/L, temperature 298 K, and equilibration time 90 min. For reusability experiments, Liposome@PDA microspheres were isolated by centrifugation after MB adsorption, rinsed three times with methanol by sonication for 5 min, rinsed with ultrapure water, and reused for the next adsorption process. The experiments were carried out in triplicate and the results normalized with respect to a reference test carried out in pure water and in optimized conditions. Adsorbent mass was 0.06 mg, MB concentration 4 mg/L, temperature 298 K, pH = 8.0, and equilibration time 90 min.

## 4. Conclusions

In order to obtain a new material with improved adsorption performances for efficient removal of MB from water solutions, Liposome@PDA microspheres were successfully prepared by using liposomes as templating agents. Owing to their unique structure, Liposome@PDA microspheres showed very high specific surface area and exhibited outstanding adsorption performances. Indeed, compared to other PDA-based adsorbent materials reported in literature, this Liposome@PDA adsorbent showed integrated high adsorption rate and adsorption capacity, which reached a high maximum adsorption capacity of 395.4 mg g^−1^ at 298 K. Moreover, the adsorption process agreed well with the pseudo-second-order kinetic model and Langmuir adsorption isotherm model, indicating that the adsorption is controlled by chemisorption.

Finally, adsorption experiments in model effluent showed that the prepared microspheres kept good adsorption performance in complex solutions, while reusability experiments with three adsorption-desorption cycles demonstrated an excellent reusability of the adsorbent system.

The achieved Liposome@PDA microspheres therefore performed promisingly in wastewater treatment for dye pollutants removal and can represent a model approach, opening up novel routes for the development of PDA-based adsorbent materials for water remediation.

## Figures and Tables

**Table 1:** Comparison of adsorption capacities of different PDA-based adsorbents for MB removal at 298 K.

PDA-Based Adsorbents	q_max_ (mg g^−1^)	Refs.
PDA-porous silica microspheres (separation in fixed bed)	83.8	[5]
Acid etching coconut shells carbon -PDA	79.32	[6]
SiO_2_-PDA-polyacrylic acid	150.02	[29]
PDA microspheres	90.7	[2]
PAAM/PA/PDA hydrogel	350.67	[34]
PDA-functionalized graphene–Fe_3_O_4_ magnetic composites	365.39	[36]
Fe_3_O_4_@PDA-Ag hollow microspheres	102.04	[37]
Fe_3_O_4_/PDA	204.1	[38]
Deacetylated cellulose acetate@PDA nanofiber membrane	88.2	[39]
Fe_3_O_4_/PDA-Fe^3+^	361.8	[40]
Liposome@PDA microspheres	395.4	This work

**Table 2:** Kinetic parameters of the models shown in Figure 6.

Kinetic Model	T (K)	q_e, exp_ (mg g^−1^)	q_e, calc_ (mg g^−1^)	k_1_ (min^−1^)	k_2_ (g mg^−1^ min^−^^1^)	k_i1/2_ (g mg^−1^ min^−0.5^)	R^2^
Pseudo-first order	298	395.36	37.60	0.047	-	-	0.8391
Pseudo-second order	298	395.36	393.70	-	0.0104	-	0.9999
Intraparticle diffusion	298	-	-	-	-	K_i1_ = 33.05 K_i2_ = 3.20	R_1_^2^ = 1.0000 R_2_^2^ = 1.0000
Intraparticle diffusion	313	-	-	-	-	K_i1_ = 72.51 K_i2_ = 4.12	R_1_^2^ = 0.8483 R_2_^2^ = 0.6481

**Table 3:** Isotherm parameters for MB adsorption onto Liposome@PDA microspheres at 298 K.

Isotherms.	Parameters	R^2^
Langmuir	q_0_ = 414.94 mg g^−1^ K_L_ = 20.25 L mg^−1^ R_L_ = 0.1220–0.0366	0.9772
Freundlich	K_F_ = 422.93 (mg g^−1^) (mg L^−1^)^n^ *n* = 4.73	0.5899

**Table 4:** Thermodynamic parameters of the adsorption of MB onto Liposome@PDA.

T (K)	K_α_ (L g^−1^)	ΔG° (kj mol^−1^)	ΔH° (kj mol^−1^)	ΔS° (kj mol^−1^ T^−1^)
278	124.56	−11.15	13.37	87.84
288	146.32	−11.94		
298	158.39	−12.55		
313	242.89	−14.29		

## Supplementary Materials

The following are available online at https://www.mdpi.com/article/10.3390/ijms222111916/s1.
