# Supplementary material for: Easy Preparation of Liposome@PDA Microspheres for Fast and Highly Efficient Removal of Methylene Blue from Water
Source: Int J Mol Sci. 2021 Nov 3;22(21):11916. doi: 10.3390/ijms222111916 (PMC8584841; doi:10.3390/ijms222111916)
Supplement: Supplementary file 1 [file ijms-22-11916-s001.zip › ijms-1434456-supplementary.pdf]

Supporting information

Easy preparation of Liposome@PDA microspheres for fast and highly efficient removal of methylene blue from water

Vincenzo De Leo [1,*], Anna Maria Maurelli [1], Chiara Ingrosso [2], Fabio Lupone [1] and Lucia Catucci [1,*]

[1] Department of Chemistry, University of Bari, Via Orabona 4, 70126 Bari, Italy

[2] CNR-IPCF S.S. Bari, c/o Dept. of Chemistry, University of Bari, Via Orabona 4, 70126 Bari, Italy

* Correspondence: vincenzo.deleo@uniba.it; lucia.catucci@uniba.it

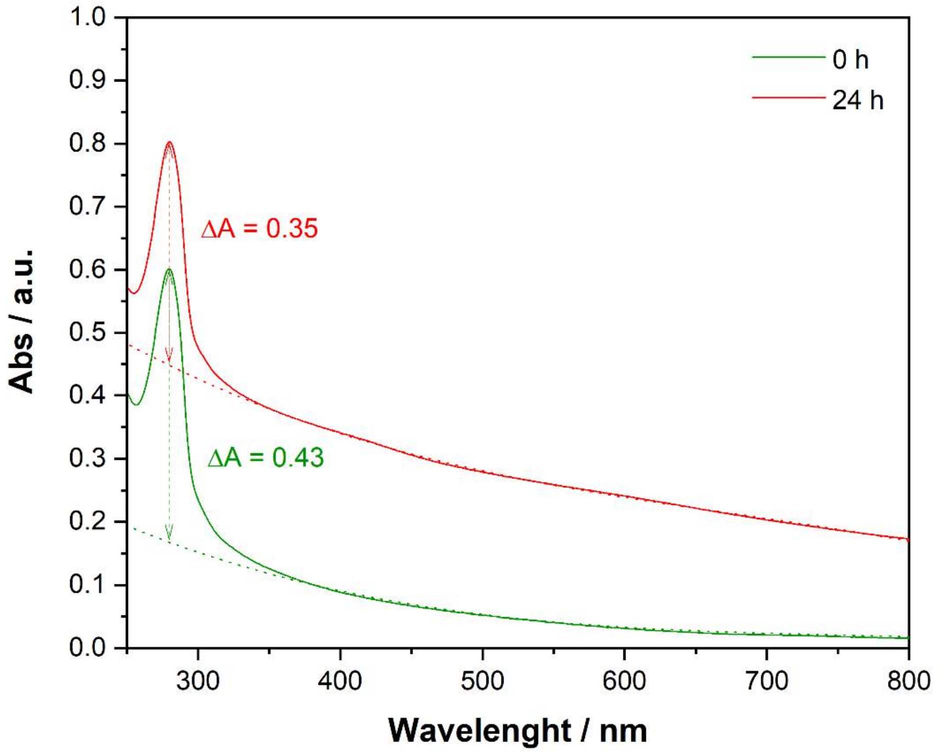

Figure S1: The characteristic absorption peak of the DA at 280 nm, which decreases as the polymerization proceeds. To subtract the contribution of the scattering due to liposomes (at 0

h) and Liposome@PDA microspheres (at 24 h), scattering was estimated by interpolating the traces with a third-degree polynomial curve.

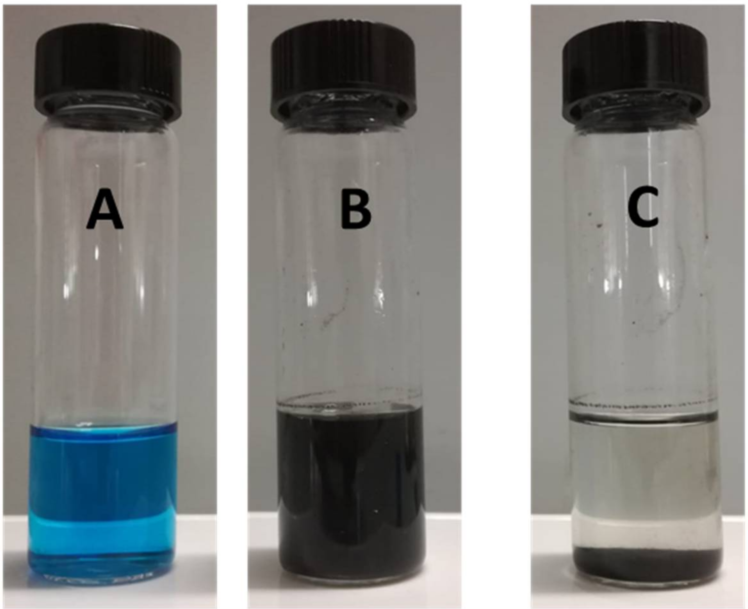

Figure S2: Stages of the adsorption process of MB by the Liposome@PDA microspheres. (A) Initial dye solution; (B) after adding Liposome@PDA adsorbent material; (C) at the end of the adsorption after sedimentation of the Liposome@PDA microspheres.

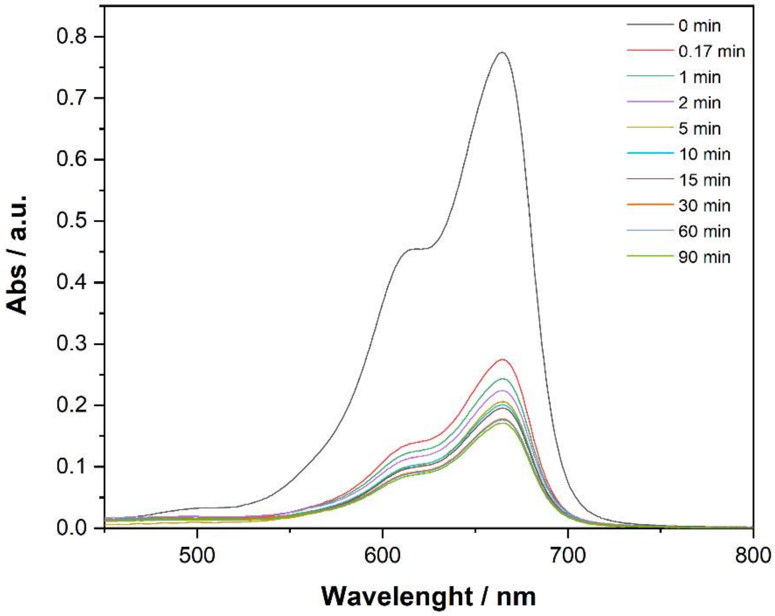

Figure S3: Vis-NIR absorption spectra of MB recorded during a typical experiment of batch adsorption monitored over time. MB initial concentration: 4 mg L^{-1}. Liposome@PDA microspheres: 0.06 mg

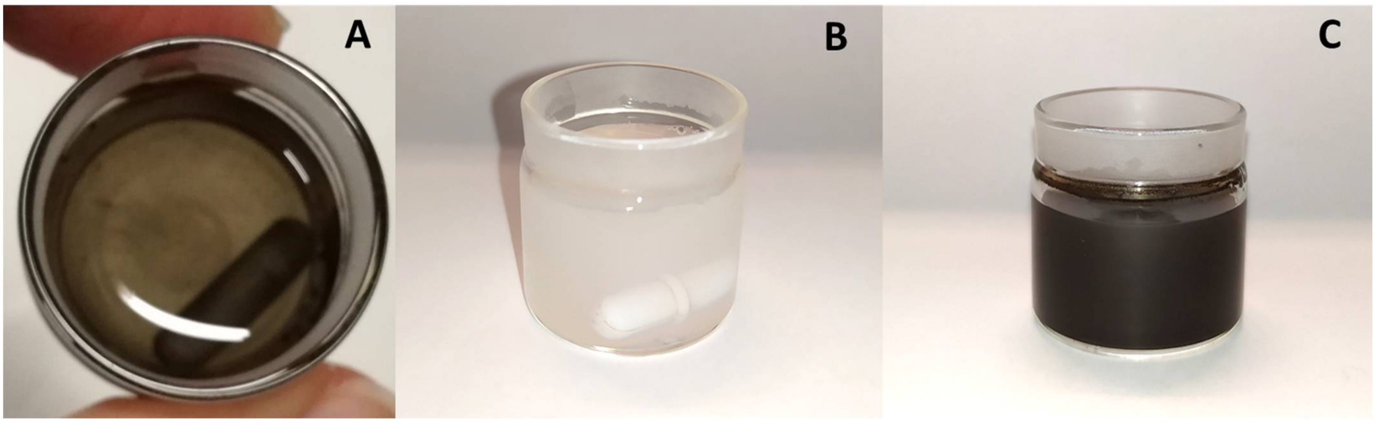

Figure S4: (A) In absence of liposomes, PDA polymerization occurred mainly on the vessel walls. (B) Liposomal suspension in DA solution at zero polymerization time. (C) After 24 h of polymerization the solution became dark indicating the formation of Liposome@PDA microspheres. In all samples, the initial DA concentration was 0.5 mg mL^{-1}.
